# Neural effects of acute stress on appetite: A magnetoencephalography study

**DOI:** 10.1371/journal.pone.0228039

**Published:** 2020-01-22

**Authors:** Chika Nakamura, Akira Ishii, Takashi Matsuo, Rika Ishida, Takahiro Yamaguchi, Katsuko Takada, Masato Uji, Takahiro Yoshikawa

**Affiliations:** Department of Sports Medicine, Osaka City University Graduate School of Medicine, Abeno-ku, Osaka, Japan; University of British Columbia, CANADA

## Abstract

Stress is prevalent in modern society and can affect human health through its effects on appetite. Therefore, in the present study, we aimed to clarify the neural mechanisms by which acute stress affects appetite in healthy, non-obese males during fasting. In total, 22 volunteers participated in two experiments (stress and control conditions) on different days. The participants performed a stress-inducing speech-and-mental-arithmetic task under both conditions, and then viewed images of food, during which, their neural activity was recorded using magnetoencephalography (MEG). In the stress condition, the participants were told to perform the speech-and-mental-arithmetic task again subsequently to viewing the food images; however, another speech-and-mental-arithmetic task was not performed actually. Subjective levels of stress and appetite were then assessed using a visual analog scale. Electrocardiography was performed to assess the index of heart rate variability reflecting sympathetic nerve activity. The findings showed that subjective levels of stress and sympathetic nerve activity were increased in the MEG session in the stress condition, whereas appetite gradually increased in the MEG session only in the control condition. The decrease in alpha band power in the frontal pole caused by viewing the food images was greater in the stress condition than in the control condition. These findings suggest that acute stress can suppress the increase of appetite, and this suppression is associated with the frontal pole. The results of the present study may provide valuable clues to gain a further understanding of the neural mechanisms by which acute stress affects appetite. However, since the stress examined in the present study was related to the expectation of forthcoming stressful event, our present findings may not be generalized to the stress unrelated to the expectation of forthcoming stressful event.

## Introduction

Stress is prevalent in modern life and society [1–3] Stress can be defined as a state in which homeostasis is threatened or perceived as threatened [4, 5]. Responses induced by stressors (i.e., stress responses) such as adverse life events can affect homeostasis physically and/or emotionally. An stress response as adaptive responses to re-establish homeostasis is necessary for maintaining homeostasis and well-being, but an excessive and/or prolonged stress response can lead to behavioral and somatic pathological conditions [4]. It has been reported that stress can affect health not only through direct biological effects [6], but also through alterations in healthy behavior such as changes in diet and appetite [6, 7]. Therefore, to prevent and reduce the adverse effects on health caused by stress, it is of great importance to clarify the neural mechanisms by which stress affects appetite.

It is thought that the suppression of appetite is caused through mechanisms such as increased glycogenolysis and delayed gastric emptying induced by the secretion of adrenaline, corticotropin-releasing hormone, and α-melanocyte-stimulating hormone, at least in acute stress [5, 6, 8]. In fact, the suppression of food intake has been reported to be induced by acute stress in rats [9, 10]. Although several studies have investigated the alteration of food intake caused by acute stress in humans [11, 12], knowledge about the alteration of subjective appetite caused by acute stress in humans remains limited. For example, in a previous study in which the level of satiety just after performing a unsolvable task (i.e., stressful cognitive task) was compared with that just after performing a solvable task (i.e., non-stressful cognitive task) [13], a decrease in satiety caused by performing the stressful task was observed: Satiety is defined as the feeling of fullness that persists after eating, suppressing further consumption and satiation is involved in the control of appetite, limiting energy intake [14, 15]. This observation implies that the increase in appetite was induced by the stressful task; however, this seems to be contradictory to the expectation that acute stress suppresses appetite as discussed above. In their study, since the cognitive demand of the task used to induce stress (i.e., the unsolvable task) was not comparable to that used not to induce stress (i.e., the solvable task), the difference in the cognitive load between the two tasks may have had some effects on the levels of satiety, resulting in the observation that the increase in appetite was caused by the stress. Therefore, it is thought to be important to control the cognitive load of the task which induces acute stress when assessing whether stress suppress appetite or not.

It has also been proposed that the control of appetite through networks in the forebrain and brain stem, which are related to the processing of food rewards, plays an important role in regulating food intake (i.e., non-homeostatic control of appetite), especially in stressful situations [13]. Non-homeostatic appetite control is an important factor in regulating food intake in humans [16, 17]. In addition, since food cues are abundant in modern environments and affect food-related behaviors [18], it is of great value to clarify the neural effects of stress on non-homeostatic appetite control in terms of neural responses to visual food cues [19–22].

In the present study, we aimed to clarify the neural mechanisms by which acute stress affects appetite in healthy normal-weight males during fasting, with a focus on stress caused by expecting critical personal events such as school examinations and public speaking engagements. To exclude the effects caused by the differences between the tasks used to and not to induce stress, such as cognitive load, on the subjective level of appetite, we designed our experiments so that the tasks participants performed in a condition with stress (i.e., the stress condition) was identical to that performed in a condition without stress (i.e., the control condition): Our participants performed the identical stress-inducing task both in the stress and control conditions. Although this could lead to the increase of the level of stress in both conditions, by instructing our participants that they were to perform another stress-inducing session later in the experiment only in the stress condition, we aimed to keep the level of stress high after the stress-inducing task in the stress condition, compared with that in the control condition. In addition to that the cognitive load in the stress condition was the same as that in the control condition, this procedure to induce stress was beneficial for assessing the effects of stress related to the expectation of the critical personal events on appetite, which was the aim of our present study. Magnetoencephalography (MEG) was used to record neural activity and to detect changes in oscillatory power reflecting neural dynamics [23] during the presentation of food images after the stress-inducing tasks both in the stress and control conditions. Since it has been reported that a decrease of alpha band power is related to the processing of sensory or cognitive information [23–25], we focused on the alteration in alpha band (8–13 Hz) power caused by viewing the food images in our present study. Clarifying the alteration in alpha band power caused by our experimental procedure would be beneficial for speculating possible neural mechanisms by which stress affects appetite and conceiving further studies to examine functional relationships among neural activity related to stress and appetite. In addition, an index of heart rate variability (low-frequency [LF] component power/high-frequency [HF] component power; LF/HF ratio) was assessed during the presentation of food images because an increase in this measure has been reported to be related to physiological states such as stress [26, 27] and a variety of others [28–30]: It has been reported that the increase of the LF/HF ratio was observed in the situation of mental stress, suggesting the index of heart rate variability can be a sensitive measure of mental stress [26].

## Materials and methods

### Participants

In total, 22 healthy male volunteers (mean age ± standard deviation [SD]: 22.8 ± 1.9 y) participated in our experiment. Current smokers, individuals with a history of mental illness or brain injury, and individuals taking chronic medications that affect the central nervous system were excluded. None of the participants were obese (body mass index: 22.7 ± 2.7 kg/m^2^). All participants were right-handed according to the Edinburgh Handedness Inventory [31]. The study protocol was approved by the Ethics Committee of Osaka City University (approval number: 3788), and written informed consent was obtained from all participants in accordance with the principles of the Declaration of Helsinki.

### Experimental design

**Fig 1:**
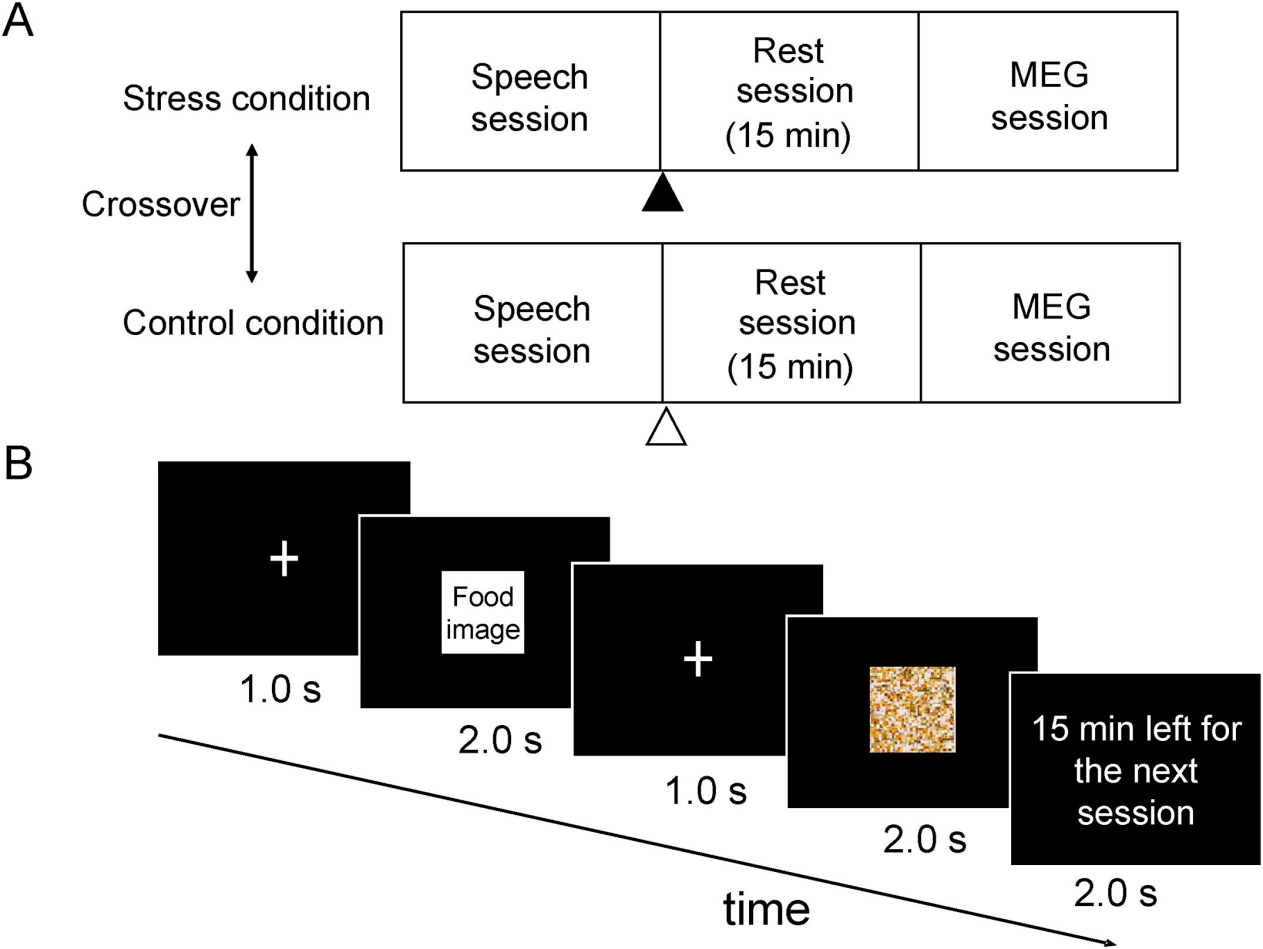
Experimental design. **(A) This study consisted of two conditions: a stress and a control condition**. Each condition was conducted on a different day in a two-crossover fashion. The mean interval between the two experimental days was approximately 1 week. Both conditions consisted of a speech session (i.e., a speech and mental arithmetic tasks), a 15-min rest session, and a magnetoencephalography (MEG) session. After the speech session under the stress condition, the participants were instructed to perform “another speech session” after the MEG session because their achievement in the speech session was poor (▲). On the other hand, under the control condition, they were instructed to fill out a simple questionnaire after the MEG session (Δ). (B) The visual stimulus presented during the MEG session consisted of a fixation cross for 1,000 ms followed by food or mosaic images for 2,000 ms. This visual presentation sequence was played 260 times with the food and mosaic image presented randomly (i.e., the food and mosaic images were each presented 130 times). The time remaining before the start of the next session was presented for 2,000 ms approximately every 78 s so that the participants would remember that they had to perform another session (i.e., “another speech session” under the stress condition and a “questionnaire session” under the control condition).

This study consisted of two conditions (i.e., a stress condition and a control condition), each of which was conducted on a different day in a two-crossover fashion (Fig 1A). The mean interval between the two experimental days was approximately 1 week. The participants were asked to fast from 9:00 pm on the day before each experimental day until the end of the experiment (they were permitted only to drink water), to avoid intense exercise and mental activity, and to maintain their usual sleeping hours. Under both the stress and control conditions, they performed a speech session, a rest session, and an MEG session. The speech session consisted of a speech and mental arithmetic tasks. They were requested to give a 3-min speech on a topic given at the time and then to count backwards from a given number (seed number) by repeatedly subtracting 13 or 14, depending on the seed number, from the number acquired most recently for 3 min. Two topics (“Describe the things you can do to prevent global warming” and “Describe your hometown”) and two seed numbers (2097 and 2083) for the sequential subtraction were prepared, and then the topics and seed numbers were randomly assigned to the stress and control conditions. The participants were told that their speech and performance of the mental arithmetic task would be evaluated by an interviewer. Under the stress condition, they were instructed to perform “another speech session” after the MEG session because their achievement in the speech session was poor, just after the speech session. On the other hand, under the control condition, they were instructed to fill out a simple questionnaire after the MEG session (i.e., questionnaire session). However, in reality, they did not perform “another speech session” in the stress condition. After a 15-min rest session, they lay in a supine position on a bed in a magnetically shielded room and viewed a visual stimulus projected on a screen by a video projector (PG-B10S; SHARP, Osaka; MEG session). The visual stimulus presented in the MEG session consisted of a fixation cross for 1,000 ms, followed by food or mosaic images for 2,000 ms (Fig 1B). This visual presentation sequence was played 260 times, with the food and mosaic image presented randomly (i.e., the food and mosaic images were each presented 130 times). The participants were instructed to have appetitive motives for each food item as if they brought each food item to their own mouth every time when the food images were presented [19]. They were also instructed not to recall their past experiences related to the food items or imagine the taste of the food items. During the visual presentation, the time remaining before the start of the next session (i.e., “another speech session” under the stress condition and the “questionnaire session” under the control condition) was presented for 2,000 ms approximately every 78 s (i.e., nine times across the MEG session) so that the participants would remember that they had to perform “another speech session” or the “questionnaire session” after the MEG session under the stress and control conditions, respectively. We used pictures of typical Japanese food items as food images [32, 33], whereas the mosaic images were created from each food image as control images using commercial software (Adobe Photoshop Elements 6.6; Adobe Systems Inc., San Jose, CA) (Yoshikawa et al., 2013; Takada et al., 2018). The set of food and control images used in our present study was identical to those used in our previous study [22]. The participants were asked to rate their subjective levels of stress and appetite on a 100-mm visual analog scale (VAS) just before and after the speech and MEG sessions under each condition. We asked our participants whether they could maintain the fasting as they were instructed in a form of questionnaire on each experimental day.

The experiments were started between 9:30 AM and 3:30 PM. Among the 20 participants whose data were analyzed in our present study (please refer to the Results), 10 participants started both the stress and control conditions exactly at the same time of the day. As for the rest 10 participants, the time of the day at which the stress condition started was later than that at which the control condition started for 5 participants and the time of the day at which the control condition started was later than that at which the stress condition started for the other 5 participants.

### MEG recording

As originally described in [34], MEG recording was performed using a 160-channel whole-head-type MEG system (MEG Vision; Yokogawa Electric Corporation, Tokyo, Japan) with a magnetic field resolution of 4 ft/Hz^1/2^ in the white-noise region. The sensors and reference coils were gradiometers with a 15.5-mm diameter and 50-mm baseline, and the two coils were separated by 23 mm. The sampling rate was 1,000 Hz, and data were high-pass filtered at 0.3 Hz.

### MEG analysis

The analyses of the MEG data were performed by a similar method to that of our previous studies. As described in [34], the magnetic noise that originated from outside of the magnetically shielded room was eliminated by subtracting the data obtained from the reference coils using specialized software (MEG 160; Yokogawa Electric Corporation) before processing the MEG data. Epochs of the raw MEG data that included artifacts were visually identified and excluded from the analyses before averaging. To identify the changes in oscillatory neural activity related to viewing the food and mosaic images, spatial filtering analysis of the MEG data was performed. The MEG data were band-pass filtered at 8–13 Hz by a finite impulse response filtering method using Brain Rhythmic Analysis for MEG software (BRAM; Yokogawa Electric Corporation) to obtain alpha band signals. After the band-pass filtering, the location and intensity of the cortical activities were estimated using BRAM, which uses a narrow-band adaptive spatial filtering algorithm. The voxel size was set at 5.0 × 5.0 × 5.0 mm, and the oscillatory power of the MEG data in the time window of 250 ms from 0–2,000 ms after the onset of the picture presentation was calculated relative to that in the time period –500 to 0 ms from the onset of the picture presentation (i.e., baseline).

These data were then analyzed using statistical parametric mapping (SPM8, Wellcome Department of Cognitive Neurology, London, UK) implemented in Matlab (MathWorks, Natick, MA, USA). The magnetic resonance (MR) image were transformed into the Montreal Neurological Institute (MNI) T1-weighted image template and then, the MEG data were transformed using the identical parameters used to transform the MR images into MNI template, using SPM. The anatomically normalized MEG data were filtered with a Gaussian kernel of 20 mm (full-width at half-maximum) in the x-, y-, and z-axes. To enable inferences to be made at the population level, individual data were summarized and incorporated into a random-effects model. The weighted sum of the parameters estimated in the individual analysis was used to create “contrast” images. The contrast images were then analyzed in a flexible factorial design with pictures (i.e., food or mosaic images) and tasks (i.e., stress or control tasks) as within-subject factors and the interaction corresponding to the contrast between “stress (food—mosaic)” and “control (food—mosaic)” was calculated for each time window. The significance of the interaction was assessed on a voxel-by-voxel basis. The threshold for the analysis was set at *P* < 0.00625 (corrected for multiple voxel-wise comparisons using familywise error rate on SPM), considering the multiple comparisons among time windows (i.e., eight time windows). In other words, we corrected for multiple comparisons within each time window using SPM’s correction tool, setting alpha to 0.00625 which is corrected for multiple voxel-wise comparisons with familywise error rate. The statistical threshold of the statistical parametric map in our figure was set at *P* < 0.05 (family-wise error-corrected for multiple comparisons) for the purpose of presentation. The resulting set of voxel values for each comparison constituted a statistical parametric map (SPM) of the statistic (SPM{t}). The SPM{t} was transformed into the units of normal distribution (SPM{Z}). The brain region was identified using WFU_PickAtras, Version 3.0.4 (http://fmri.wfubmc.edu/software/pickatras) and Talairach Client, Version 2.4.3 (http://www.talairach.org/client.html).

### Magnetic resonance (MR) image overlay

Anatomical MR imaging (MRI) was performed using a whole-body 3.0 T scanner (Philips Achieva 3.0 TX; Royal Philips Electronics, Eindhoven, The Netherlands) to permit registration of magnetic source locations with their respective anatomical locations. Before the MR imaging, five adhesive makers (Medtronic Surgical Navigation Technology Inc., Broomfield, CO, USA) were attached to the skin of the scalp: two markers 10 mm in front of the left and right tragus, one marker 35 mm above the nasion, and two markers 40 mm to either side of the marker above the nasion. The MEG data were then superimposed on MR images using information obtained from these markers and the MEG localization coils: In the MEG sessions, five MEG localization coils were attached to the identical points on the skin of the scalp where the adhesive makers for the MR imaging was placed and the locations of these localization coils were measured during the MEG recordings.

### Electrocardiography (ECG)

To examine the changes in autonomic nerve activity in the MEG session caused between the stress and control conditions, electrocardiography (ECG) was performed during the MEG recordings. The ECG data were recorded, transferred to the MEG system, and analyzed with the maximum entropy method using MemCalc for Windows (Global Medical Solution Inc., Tokyo, Japan). R-R wave variability was measured as an indicator of autonomic activity. For frequency domain analysis of the R-R wave interval, LF power was calculated as that within the frequency range of 0.04–0.15 Hz, and HF power as that within the frequency range of 0.15–0.4 Hz. LF and HF power were measured in absolute units (ms^2^). It has been reported that HF power is vagally mediated [35–37], whereas LF power originates from a variety of sympathetic and vagal mechanisms [35, 38]. The LF/HF ratio is considered an index of sympathetic nerve neurons system activity. The natural logarithms of LF, HF, and LF/HF were calculated and used for the statistical analyses (i.e., ln LF, ln HF, and ln LF/HF).

### Statistical analysis

Values are presented as the mean ± SD unless otherwise stated. Two-way analysis of variance (ANOVA) with repeated measures was performed to assess the effect of the stress and control conditions on subjective levels of appetite. A paired *t* test with Bonferroni’s correction was used to compare the subjective levels of stress and appetite, and indexes of autonomic nerve function such as LF, HF, and LF/HF just before and after the MEG session compared with those before the speech session. All *P* values were two-tailed and *P* < 0.05 was considered statistically significant. All statistical analyses mentioned above were performed using SPSS (version 21.0; IBM, Armonk, NY, USA).

## Results

### Subjective and objective levels of stress

**Fig 2:**
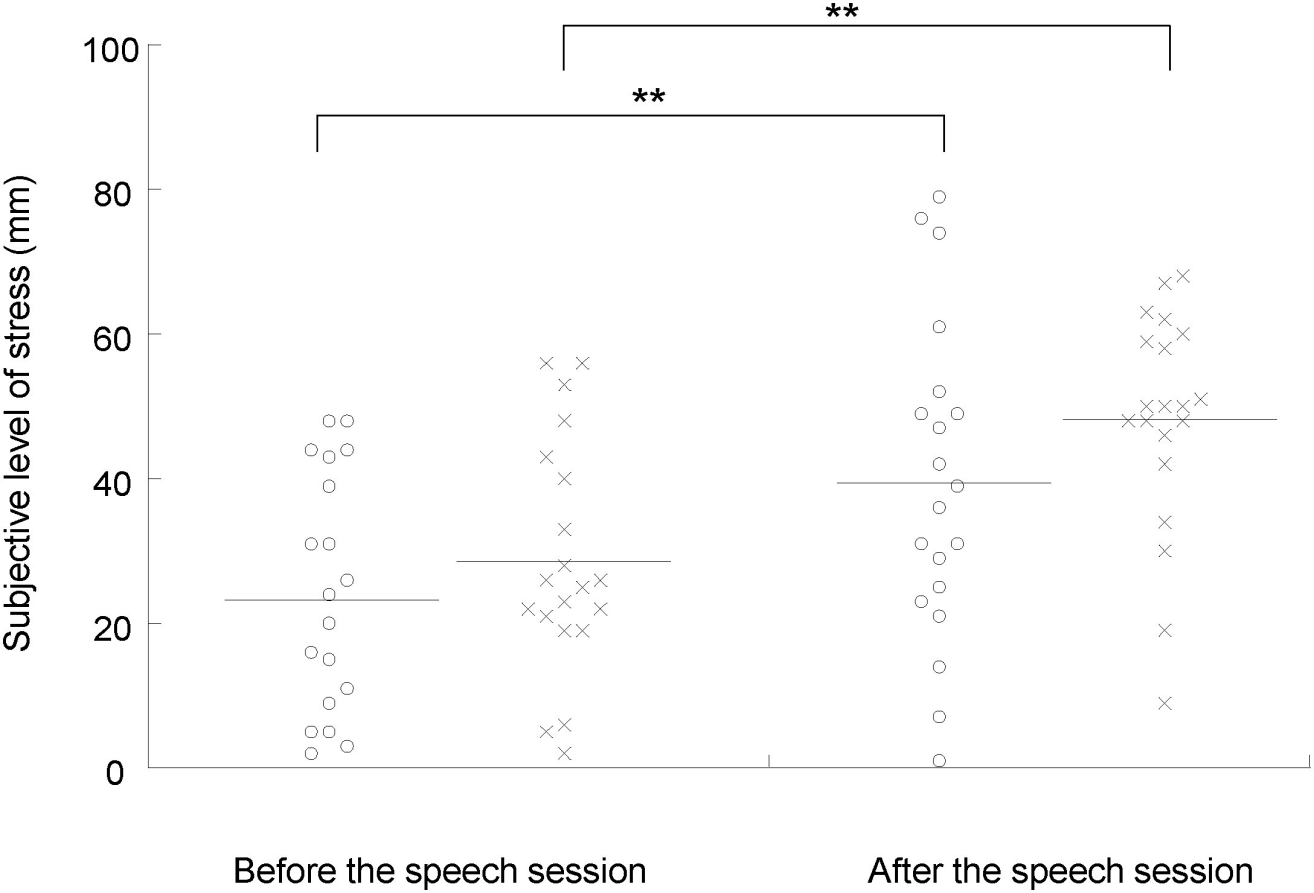
Subjective levels of stress before and after the speech sessions under the stress (open circles) and control (crosses) conditions. Participants were asked to rate their subjective level of stress on a 100-mm visual analog scale (VAS) from 0 (minimum stress) to 100 (maximum stress). The horizontal line in each plot indicates mean value. **P < 0.01, paired t test with Bonferroni’s correction.

**Fig 3:**
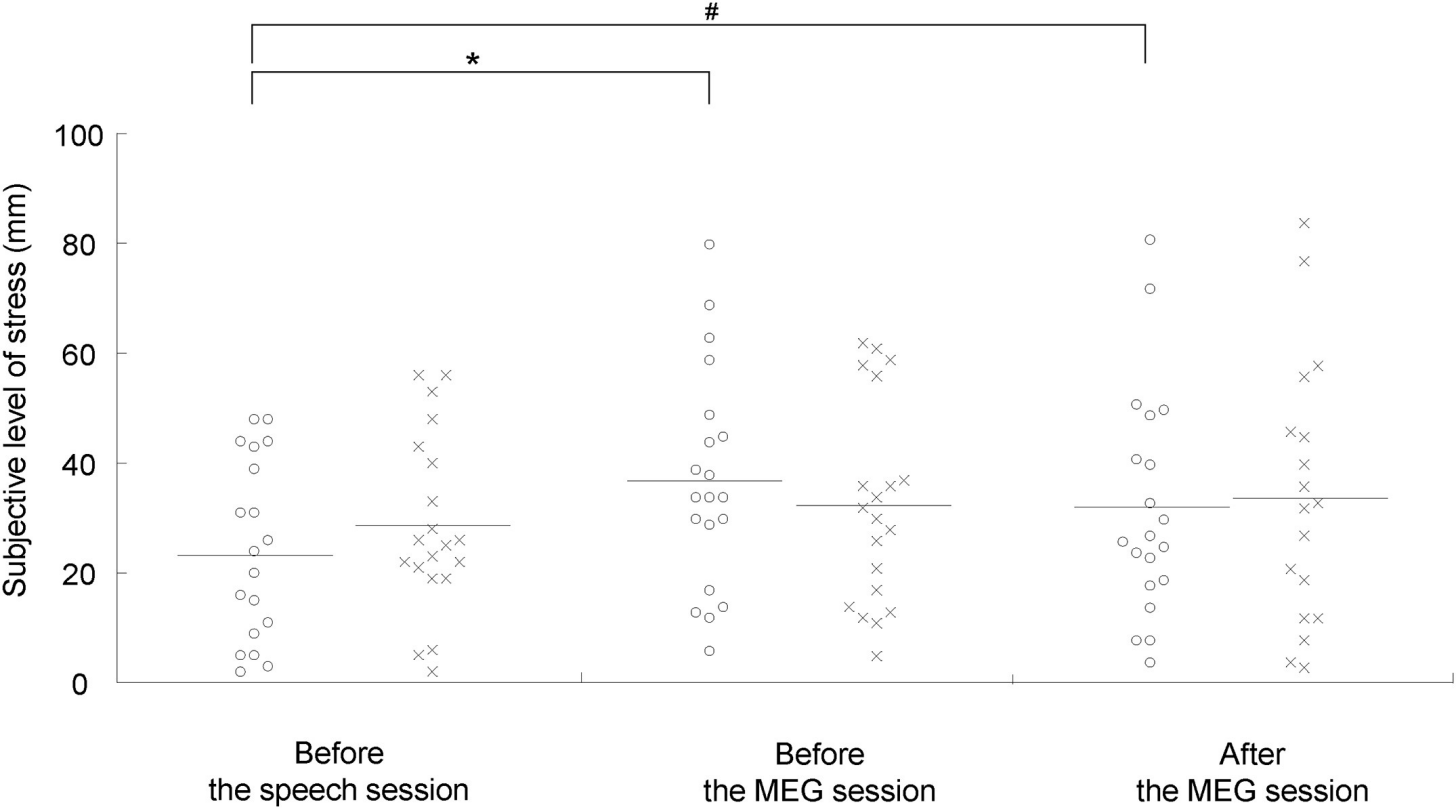
Subjective levels of stress before the speech session and before and after the magnetoencephalography (MEG) sessions under the stress (open circles) and control (crosses) conditions. Participants were asked to rate their subjective level of stress on a 100-mm visual analog scale (VAS) from 0 (minimum stress) to 100 (maximum stress). The horizontal line in each plot indicates mean value. *P < 0.05 and #*P* < 0.10, paired t test with Bonferroni’s correction.

**Fig 4:**
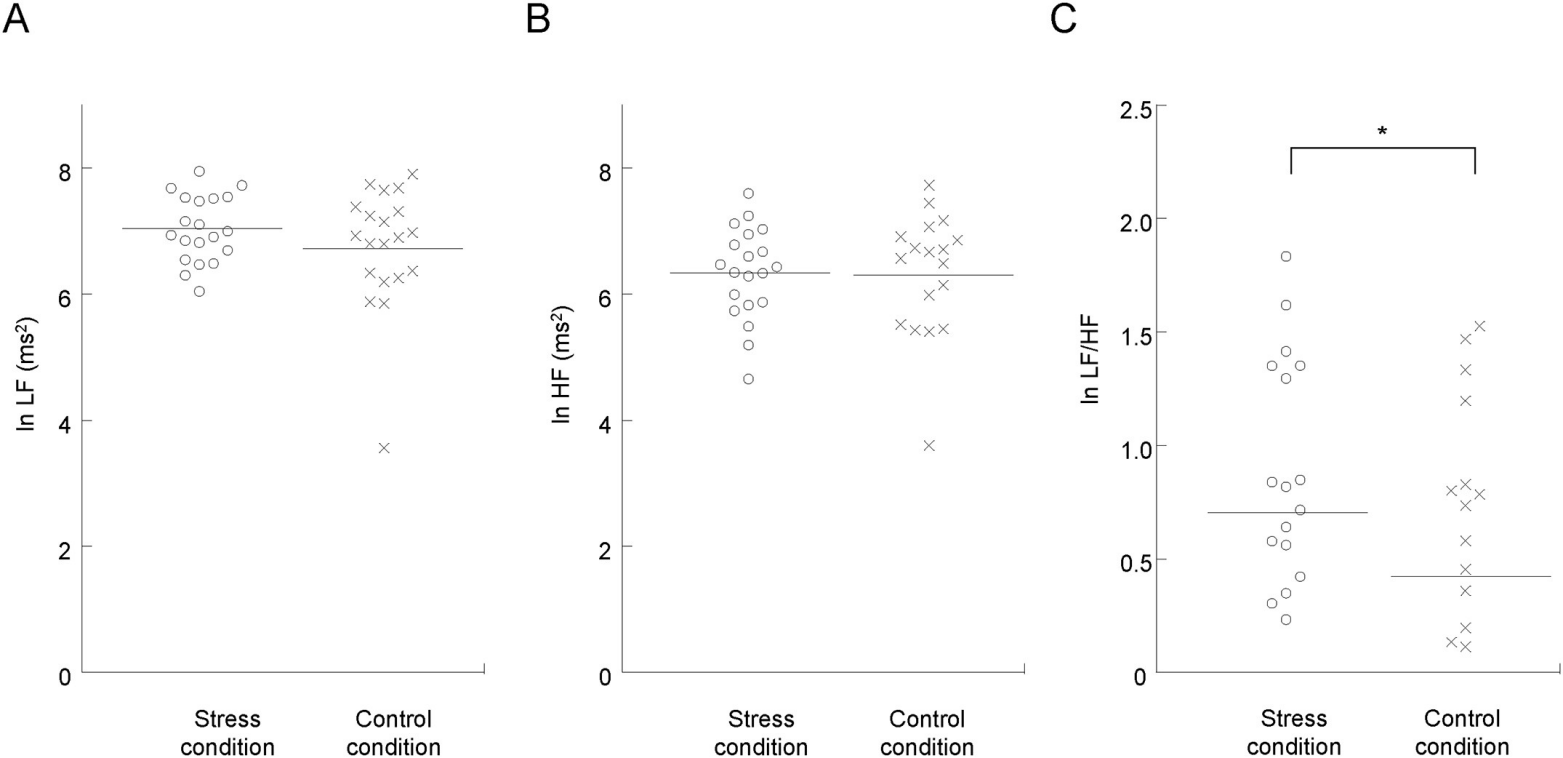
Alterations in autonomic activity as assessed by frequency analysis of R-R wave intervals of electrocardiography during the magnetoencephalography (MEG) session under the stress (open circles) and control (crosses) conditions. Values were transformed by natural logarithm (ln). Low-frequency power (ln LF; A), high-frequency power (ln HF; B), and the LF/HF ratio (ln LF/HF; C) under the stress and control conditions are shown. The horizontal line in each plot indicates mean value. *P < 0.05, paired t test with Bonferroni’s correction.

The VAS scores for stress and appetite and the indices of sympathetic nerve activity were analyzed for 20 participants, because two participants failed to satisfy the requirements for participating in the study: One declared that he could not maintain the fasting and the other fell asleep during the experiments. Under both the stress and control conditions, the subjective levels of stress were higher after than before the speech session (*P* < 0.001, paired *t* test with Bonferroni’s correction; Fig 2). The subjective levels of stress just before the MEG session were higher than those before the speech session only under the stress condition (*P* < 0.05, paired *t* test with Bonferroni’s correction; Fig 3), while those after the MEG session showed a tendency to increase compared with those before the speech session under the stress condition (*P* = 0.055, paired *t* test with Bonferroni’s correction; Fig 3). The ln LF/HF ratio assessed during the MEG session was increased under the stress condition compared with that under the control condition (*P* < 0.05, paired *t* test with Bonferroni’s correction; Fig 4). The means and SDs of the subjective levels of stress and those of the LF, HF, and LF/HF ratio were summarized in Tables 1 and 2, respectively. The alteration of the LF/HF ratio between the stress and control conditions was not correlated with the difference in the start time of the experiments between the stress and control conditions (*P* = 0.643, r = -0.110; Pearson’s correlation analysis).

**Table 1:** Means and SDs of the subjective levels of stress before and after the speech and MEG sessions.

	Before the speech session (mm)	After the speech session (mm)	Before the MEG session (mm)	After the MEG session (mm)
Stress condition				
Mean	23.2	39.3	37.0	32.2
SD	16.9	22.0	20.0	20.5
Control condition				
Mean	28.7	48.1	32.4	34.1
SD	16.2	15.4	18.4	22.6

SD, standard deviation.

**Table 2:** Means and SDs of the indices of autonomic activity as assessed by frequency analysis of R-R wave intervals in the stress and control conditions.

	LF (ms^2^)	HF (ms^2^)	LF/HF (ms^2^)
Stress condition			
Mean	7.04	6.33	0.70
SD	0.53	0.73	0.65
Control condition			
Mean	6.75	6.33	0.42
SD	0.97	0.93	0.68

SD, standard deviation.

ln, natural logarithm; LF, Low-frequency power; HF, high-frequency power; LF/HF, the LF/HF ratio.

### Subjective level of appetite

**Fig 5:**
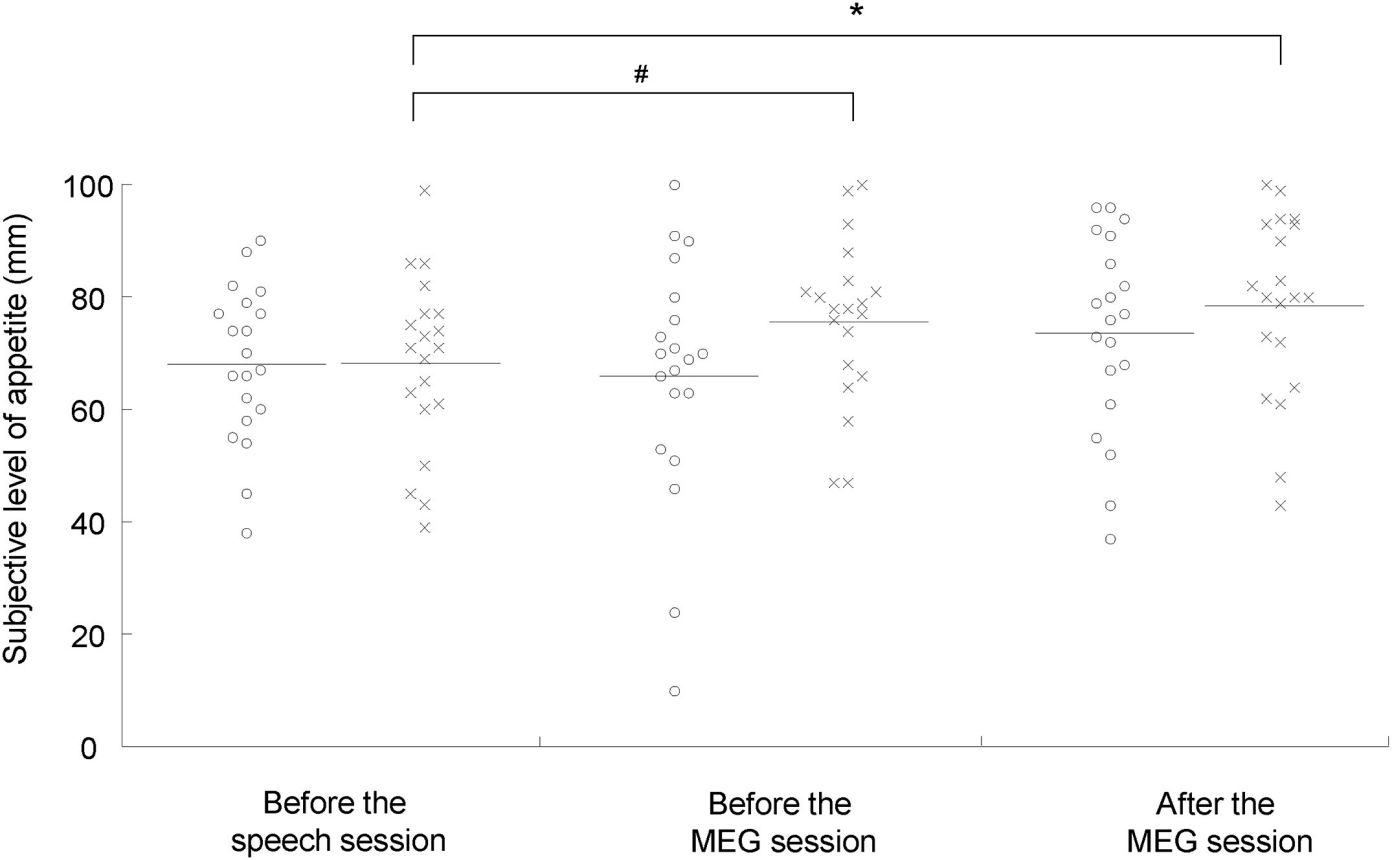
Subjective levels of appetite before the speech session and before and after the magnetoencephalography (MEG) session under the stress (open circles) and control (crosses) conditions. Participants were asked to rate their subjective level of appetite on a 100-mm visual analog scale (VAS) from 0 (minimum appetite) to 100 (maximum appetite). The horizontal line in each plot indicates mean value. *P < 0.05 and #*P* < 0.10, paired t test with Bonferroni’s correction.

To assess the effect of the stress and control conditions on subjective levels of appetite, a two-way ANOVA with repeated measures across conditions and time points was performed. Main effects of time [F(2, 38) = 4.167, *P* = 0.016] and condition × time interaction [F(2, 38) = 4.580, *P* = 0.017] were observed. The subjective level of appetite was higher after the MEG session than before the speech session under the control condition (*P* < 0.05, paired *t* test with Bonferroni’s correction; Fig 5), and that before the MEG session showed a tendency toward increase compared with that before the speech session under the control condition (*P* = 0.064, paired *t* test with Bonferroni’s correction; Fig 5). The subjective level of appetite after the MEG session was not altered compared with that before the MEG session in the stress condition (*P* = 0.26, paired t test with Bonferroni’s correction), while the subjective level of appetite after the MEG session was increased compared with that before the MEG session in the control condition (*P* = 0.047, paired t test with Bonferroni’s correction). The means and SDs of the subjective levels of appetite was summarized in Table 3. The alteration of appetite between before the MEG and before the speech sessions in the control condition was not correlated with the difference in the start time of the experiments between the stress and control conditions (*P* = 0.999, r = 0.000171; Pearson’s correlation analysis). The alteration of appetite between after the MEG and before the speech sessions in the control condition was not correlated with the difference in the start time of the experiments between the stress and control conditions (*P* = 0.725, r = -0.0838; Pearson’s correlation analysis).

**Table 3:** Means and SDs of the subjective levels of appetite before and after the speech and MEG sessions.

	Before the speech session (mm)	After the speech session (mm)	Before the MEG session (mm)	After the MEG session (mm)
Stress condition				
Mean	68.2	65.5	66.0	73.9
SD	13.8	19.3	21.7	17.3
Control condition				
Mean	68.3	70.9	75.9	78.5
SD	15.5	14.5	14.5	16.2

SD, standard deviation.

### Spatial filtering analyses of the MEG data

In addition to the MEG data from the two participants who did not satisfy the requirements for participating in the study, those from three other participants were excluded from the analyses because the numbers of the epochs of their MEG data were not sufficient for the MEG analysis after excluding the epochs contaminated with magnetic noise; in the present study, we analyzed the MEG data with more than 40 epochs. Therefore, we finally analyzed MEG data from 17 participants.

**Fig 6:**
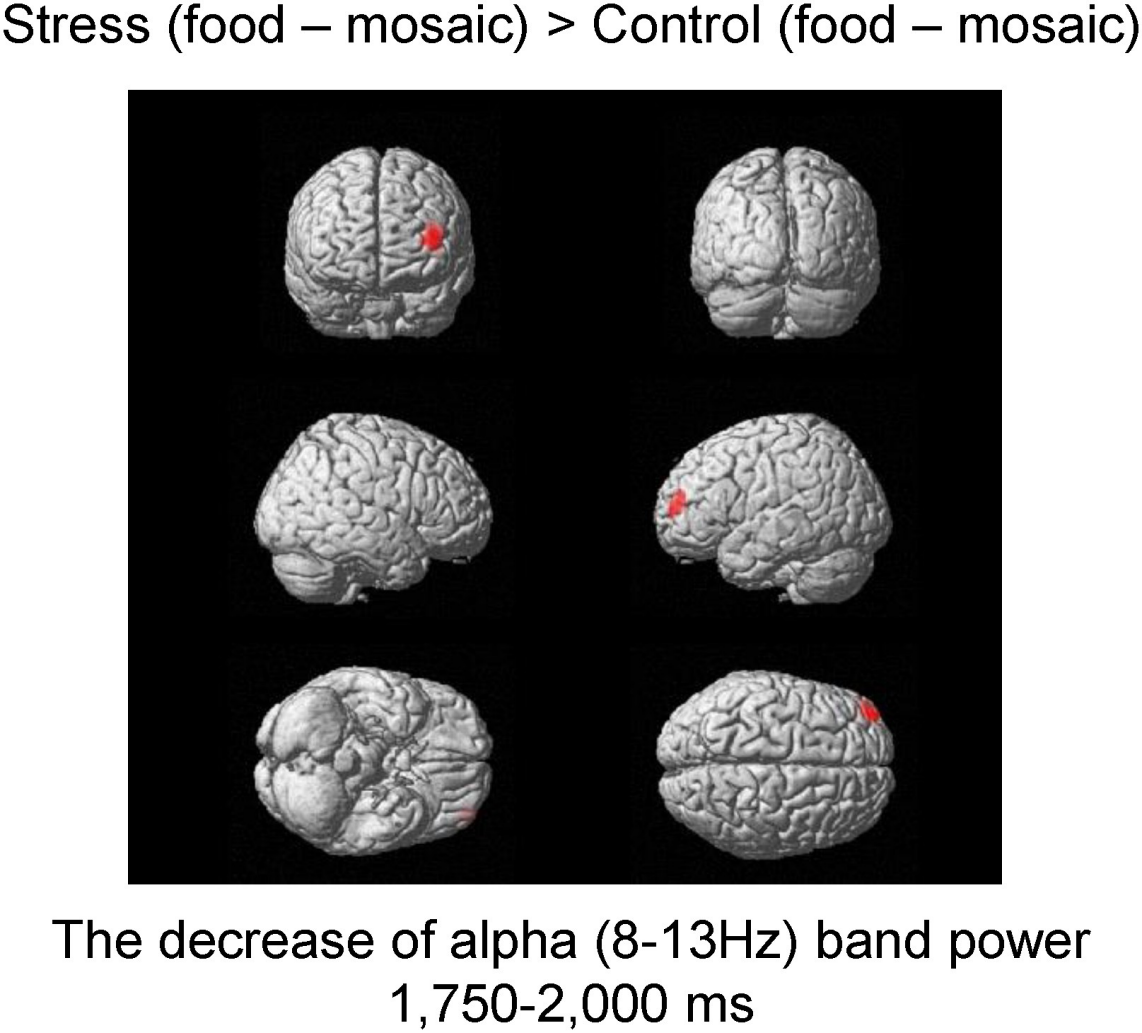
Statistical parametric maps of brain areas where the decrease of alpha band power in the frontal pole (Brodmann’s area 10) in the time window of 1750–2000 ms was higher under the stress compared with the control condition. Random-effect analyses of 17 participants, *P* < 0.05, family-wise error-corrected for the entire search volumes.

To identify the changes in neural activity caused by the experimental conditions (i.e., the stress and control conditions), the alterations of alpha-band oscillatory brain activity induced by viewing the food images in the stress condition were compared with those in the control condition. The decrease of alpha band power in Brodmann’s area (BA) 10 (i.e., the frontal pole) in the time window of 1,750–2,000 ms after the onset of the picture presentation caused by viewing the food images in the stress condition was greater than that caused by viewing the food images in the control condition (Fig 6, Table 4). The number of voxels in the significant cluster was 1 when the statistical threshold was set at *P* = 0.00625.

**Table 4:** Brain region that showed a greater decrease of alpha band oscillatory brain activity under the stress compared with the control condition.

Time window	Location	BA	MNI coordinates (mm)			Z value
			x	y	z	
1750–2000 ms	Frontal pole	10	–43	58	15	4.3

BA, Brodmann’s area; MNI, Montreal Neurological Institute.

x, y, z: Stereotaxic coordinates.

Data were obtained from random-effect analyses. Only significant changes are shown (*P* < 0.00625, family-wise error rate).

## Discussion

In the present study, participants viewed food and control images in the stress and control conditions. In the stress condition, they were instructed to perform additional speech and mental calculation tasks that were identical to those performed at the beginning of each experimental day after an MEG session, whereas in the control condition, they were not. Subjective levels of stress were increased after the speech session compared with that before the speech session both in the stress and control conditions. Subjective level of stress before the MEG session was higher than that before the speech session in the stress condition and that after the MEG session showed a tendency toward increase compared with that before the speech session in the stress condition. Sympathetic nerve activity as assessed by the LF/HF ratio during the MEG session in the stress condition was greater than that in the control condition. In the control condition, the subjective level of appetite just before the MEG session showed a tendency toward increase compared with that before the speech session, and that just after the MEG session was higher than that before the speech session. In addition, the decrease of alpha band power in the frontal pole caused by viewing the food images in the stress condition was greater than that caused by viewing the food images in the control condition.

Since subjective levels of stress after the speech session were increased compared with those before the session both in the stress and control conditions, and since the speech session performed in the control condition was identical to that performed in the stress condition, the participants in the present study were confirmed to have experienced the speech sessions as stressful, at least subjectively, under both the stress and control conditions. Furthermore, as subjective levels of stress increased and tended to increase before and after the MEG session, respectively, only in the stress condition, and an increase in sympathetic nerve activity during the MEG session was observed in the stress condition, it was confirmed that the participants viewed the food and control images with stress only in the stress condition.

A tendency toward increase and an increase in subjective levels of appetite before and after the MEG session, respectively, were observed only in the control condition. Since subjective levels of appetite increased gradually throughout the MEG session in the control condition, but not in the stress condition, the increase of the subjective levels of appetite can be considered to have been relatively suppressed during the MEG session in the stress condition compared with those in the control condition; this finding suggests that the increase of the subjective levels of appetite in the stress condition seemed to be suppressed by the stress experienced during the MEG session: Since the participants in our present study were fasted overnight, the increase of appetite over time observed in the control condition was reasonable.

In a previous study in which the effects of acute stress on appetite were examined in females, the level of satiety just after performing a unsolvable task (i.e., a stressful cognitive task) was decreased compared with that just after performing a solvable task (i.e., a non-stressful cognitive task) both under a postprandial condition, suggesting that the increase of appetite was induced by the stressful task [13]. There is a possibility that the effect of stress on appetite differs between men and women. In fact, it has been reported that viewing a stressful film is associated with reduced food intake in men, but not in women [11]. However, since it has also been reported that acute stress caused by performing an unsolvable arithmetic task increases energy intake in both men and women [12], gender differences do not seem to have been a major reason for the suppression of appetite under the stress condition observed in our present study, against the finding of the previous study that acute stress increased appetite. As described in the Introduction, there is a possibility that, in addition to the stress induced by stress-inducing task, the cognitive load of the stress-inducing task also affected the subjective level of appetite: In fact, while the cognitive demand of the task used to induce stress was not comparable to that used not to induce stress in the previous study, our experiments were designed so that the tasks participants performed in a condition with stress was identical to that performed in a condition without stress. Therefore, our findings seem to suggest that acute stress can suppresses appetite; however, the cognitive load of the task which induces the stress may be a factor that affects the level of appetite under the stressful condition.

The decrease of alpha band power in the frontal pole caused by viewing the food images under the stress condition was greater than that under the control condition. We focused on the decrease of alpha band (8–13 Hz) power caused by viewing the food images because it has been reported that a decrease of alpha band power is related to the processing of sensory or cognitive information, reflecting cortical activity [23, 24, 39]. Therefore, information processing in the frontal pole caused by viewing food images can be interpreted as being increased by the stress caused by our experimental procedure. It has been reported that the frontal pole is involved in the thinking and planning of future actions [40, 41]. Since our participants were instructed to perform another speech session after viewing pictures in the stress condition, it is plausible that they were thinking and planning of the forthcoming speech session during the MEG session under the stress condition; this may have been one of the reasons for the activation of the frontal pole under the stress condition. In addition to the thinking and planning of future actions, the frontal pole seems to be related to the motives and cognitive control of appetite. It has been reported that the alteration of appetitive motives caused by viewing food images is associated with a decrease of gamma band power in the frontal pole assessed during a resting state just after compared with just before viewing food images [42]. In a study involving patients with anorexia nervosa, activation of the frontal pole on functional MRI was observed in response to visually presented high-calorie stimuli [43]. The authors of that study noted that since the frontal pole plays a role in supervisory processes, activation of the frontal pole is associated with the cognitive control of appetite, resulting in the under-consumption of high-calorie foods that was observed in their participants. Taking this into consideration, it is speculated that the participants’ expectations of the forthcoming speech session in our present study activated the frontal pole for the thinking and planning of future actions; this activation of the frontal pole may have interfered with the regulatory processes related to appetite that were also subserved by the frontal pole, resulting in the suppression of appetite under the stress condition.

A decrease of alpha band power in the frontal pole was observed in the time window of 1,750–2,000 ms after the onset of the picture presentation. Other MEG studies have reported that the presentation of visual food cues caused an equivalent current dipole in the insular cortex approximately 300 ms after the onset of the presentation of the food cues [19], and that an alteration in theta (4–8 Hz) band power in the dorsolateral prefrontal cortex in the time window of 500–600 ms after the onset of the presentation of food images was observed when participants were asked to suppress their appetite [20]. The fact that the latency of the neural activity in the frontal pole observed in the present study, which seems to be related to the suppression of appetite, was longer than that in a previous study (i.e., in the time window of 500–600 ms after the onset of the presentation of food images), may reflect complex and time-consuming interplay between the neural substrate of appetite and that of the thinking and planning of future actions within the brain regions involving the frontal pole.

The present study did have some limitations. First, the study participants were healthy, non-obese males who fasted on each experimental day and the number of the participants whose MEG data were analyzed was relatively small (i.e., n = 17). To further the understanding of the neural mechanisms by which stress affects appetite and generalize our results, studies with larger number of participants, women, non-fasting individuals, obese individuals, and those with eating and/or stress disorders are needed. Second, stress related to the expectation of forthcoming additional speech and mental calculation tasks was examined in the present study. Therefore, our present findings may not be generalized to the stress unrelated to the expectation of forthcoming stressful event: The neural effects of stress on appetite may vary depending on the procedure used to induce stress. Third, the alteration in the amount of food intake was not assessed in this study. Since there has been a report that food intake is increased by acute stress [12], it is of great interest whether food intake could have been increased by our experimental procedure to induce stress. Fourth, some studies regarding the effects of subacute and/or chronic stress, such as stress experienced in the workplace and in daily life, on appetite and/or food-related behaviors have been conducted; therefore, it is necessary to clarify the relationships between the effects caused by acute stress and those caused by subacute and/or chronic stress in future studies. Fifth, since the duration of the Rest session was set at 15 min and other time length of the durations for the Rest session was not tested, it is difficult to assess the effects caused by the duration of the Rest session on the level of stress in our present study. However, the duration of the Rest session in our present study (i.e., 15 min) seems to have been sufficient for achieving our purpose of our present study: To increase the level of stress in the MEG session in the stress condition while that was not increased in the control condition compared with the level of stress before the initial stress-inducing session of the corresponding experimental day. Sixth, the start time of the control condition was different from that of the stress condition for 10 participants in our present study. However, since the alteration of appetite between before the MEG and before the speech sessions in the control condition was not correlated with the difference in the start time of the experiments between the stress and control conditions and the alteration of appetite between after the MEG and before the speech sessions in the control condition was not correlated with the difference in the start time of the experiments between the stress and control conditions, the alterations in the subjective levels of appetite observed before and after the MEG sessions compared with that before the speech session in the control condition was not due to the difference in the start time between the stress and control conditions. In addition, the alteration of the LF/HF ratio between the stress and control conditions was not correlated with the difference in the start time of the experiments between the stress and control conditions, suggesting that the difference in the start time between the stress and control conditions did not affect our observation that the LF/HF ratio in the stress condition was higher than that in the control condition.

In conclusion, the results of the present study indicate that acute stress can suppress the increase of appetite. In addition, the decrease of alpha band power in the frontal pole induced by viewing food images under the stress condition was greater than that under the control condition, suggesting that the activation of the frontal pole caused by the expectation of the forthcoming speech session interfered with the regulatory processes related to appetite that were also subserved by the frontal pole. These findings may provide valuable clues to gain a further understanding of the neural mechanisms by which acute stress affects appetite, and could contribute to the development of methods to prevent and reduce the adverse effects on health caused by stress.
